# Development of Conformational Antibodies to Detect Bcl-xL’s Amyloid Aggregates in Metal-Induced Apoptotic Neuroblastoma Cells

**DOI:** 10.3390/ijms21207625

**Published:** 2020-10-15

**Authors:** Alexis Gonneaud, Fatima-Zohra Fakhir, Emeline Landas, Enora Le Tallec, Elisabeth Chartier-Garcia, Christine Almunia, Alexandre Chenal, Vincent Forge, Christel Marquette

**Affiliations:** 1 Laboratoire de Chimie et Biologie des Métaux, UMR5249 Université Grenoble Alpes, CNRS, CEA, 17 rue des Martyrs, CEDEX 9, 38054 Grenoble, France; alexis.gonneaud@usherbrooke.ca (A.G.); fatimazohra.fakhir@gmail.com (F.-Z.F.); emeline66@hotmail.fr (E.L.); enora.letallec@yahoo.fr (E.L.T.); Elisabeth.CHARTIER-GARCIA@cea.fr (E.C.-G.); vincent.forge@cea.fr (V.F.); 2 Département d’immunologie et biologie cellulaire, Pavillon de recherche appliquée sur le cancer, Faculté de médecine et des sciences de la santé, Université de Sherbrooke, Sherbrooke, QC J1E 4K8, Canada; 3 Faculté des Sciences d’El Jadida, Université Chouaib Doukkali, El Jadida 24000, Maroc; 4 Laboratory «Innovative Technologies for Detection and Diagnostics» CEA-Marcoule, DRF-Li2D PRAE Marcel Boiteux, BP 1717, 30200 Bagnols-sur-Cèze CEDEX, France; christine.almunia@cea.fr; 5 Chemistry and Structural Biology Department, Institut Pasteur, UMR CNRS 3528, CEDEX 15, 75724 Paris, France; alexandre.chenal@pasteur.fr

**Keywords:** Bcl-xL, apoptosis, oxidative stress, amyloid fibers

## Abstract

Bcl-xL, a member of the Bcl-2 family, is a pro-survival protein involved in apoptosis regulation. We have previously reported the ability of Bcl-xL to form various types of fibers, from native to amyloid conformations. Here, we have mimicked the effect of apoptosis-induced caspase activity on Bcl-xL by limited proteolysis using trypsin. We show that cleaved Bcl-xL (ΔN-Bcl-xL) forms fibers that exhibit the features of amyloid structures (BclxLcf37). Moreover, three monoclonal antibodies (mAbs), produced by mouse immunization and directed against ΔN-Bcl-xL or Bcl-xL fibers, were selected and characterized. Our results show that these mAbs specifically target ΔN-Bcl-xL in amyloid fibers in vitro. Upon metal-stress-induced apoptosis, these mAbs are able to detect the presence of Bcl-xL in amyloid aggregates in neuroblastoma SH-SY5Y cell lines. In conclusion, these specific mAbs directed against amyloidogenic conformations of Bcl-xL constitute promising tools for studying, in vitro and in cellulo, the contribution of Bcl-xL in apoptosis. These mAbs may further help in developing new diagnostics and therapies, considering Bcl-xL as a strategic target for treating brain lesions relevant to stroke and neurodegenerative diseases.

## 1. Introduction

Within neurodegenerative diseases, e.g., Alzheimer’s disease (AD) or stroke-induced ischemia, multiple and complex molecular events are implicated in critical processes, such as oxidative stress, protein aggregation, and mitochondrial dysfunctions. In these pathologies, biometals play a major role in the initiation and development of lesions. They are essential elements for life that play crucial roles in healthy organs and tissue functions, such as the brain’s neurotransmitter synthesis, neural information processing, and neuronal myelination [1]. Consequently, the local dysregulation of their concentrations generates severe changes in critical biological systems, with the initiation of cascades of events leading to cell death. Increasing evidence suggests that transition-metal homeostasis, i.e., of iron, copper, and zinc, is dysregulated in neurodegenerative pathologies [2]. In fact, these transition metals are enriched in amyloid aggregates and facilitate the stimulation of the production of reactive oxygen species (ROS) [3,4,5]. In AD, such redox activity has been reported to be sustained by Aβ amyloid fibrils, found in the brains of AD patients, and to promote local deleterious oxidative damage, and inflammation-mediated and apoptotic neuronal cell death [6]. In turn, oxidative stress and ROS contribute to AD pathophysiology by influencing the amyloid precursor protein’s processing [7] and by exacerbating Aβ aggregation [8,9].

Apoptosis is an essential and critical physiological process for maintaining tissue integrity through normal homeostasis: a balance between cell proliferation and cell death. In a context of chronic ROS production, as described in slow-onset neurodegenerative diseases, this balance shifts in favor of apoptosis via chronic inflammation [10] and the dysfunction of mitochondria [11]. There are two main pathways for the induction of apoptosis: the death receptor (or extrinsic) pathway and the mitochondrial (or intrinsic) pathway. The mitochondrial pathway involves key players, the B-cell lymphoma 2 (Bcl-2) family members, which regulate apoptosis by balancing survival and cell death [12]. This protein family is classified into three groups depending on structural and functional properties: (a) pro-survival Bcl-2 proteins, including Bcl- 2, Bcl-xL, Mcl-1, and Bcl-w [13]; (b) pro-apoptotic effector proteins, i.e., Bax and Bak [14], which interact strongly with anti-apoptotic proteins [15]; and (c) BH3-only proteins, which convey signals to initiate apoptosis, including Bim, Puma, Bid, Bik, Bad, Bmf, Hrk, and Noxa [16]. In this family, Bcl-xL plays anti-apoptotic roles by preventing the oligomerization of the pro-apoptotic proteins Bax and Bak [17,18,19,20,21,22], reducing cytochrome c release [23,24,25], and regulating caspase activity [26,27]. In addition to its importance in cell survival, Bcl-xL is reported to play critical roles in neurophysiology by controlling intracellular energy metabolism and enhancing mitochondrial adenosine triphosphate (ATP) production [28,29]. Bcl-xL also promotes neurotransmission and synaptic vesicle endocytosis homeostasis [30] and neurite outgrowth and branching [31]. It prevents the loss of axons and dendrites during neurotoxic insults and supports proper synapse formation [30].

Moreover, the activity of the Bcl-xL protein is tightly controlled by interactions between the members and regulated by their post-translational modifications such as deamidation that can reverse Bcl-xL activities from anti- to pro-apoptotic [21]. Cell death processes stimulate the phosphorylation of Bcl-xL and enable the activation of caspase cleavage [32], converting Bcl-xL to a pro-apoptotic protein mimicking the BCL-2 family of proteins such as Bax, Bid, and Bcl-2 [17,33,34,35,36,37]. Full length Bcl-xL protein contains a caspase cleavage site, producing a C-terminal fragment that has Bax-like pro-apoptotic activity. The resulting protein (called ΔN-Bcl-xL) is thought to confer the cell protection against apoptosis [38,39]. It is likely that conformational rearrangements coincident with the removal of the N-terminus segment contribute to the pro-apoptotic activity of the cleaved Bcl-xL protein, especially in the brain, where Bcl-xL is highly expressed [40] and is involved in various processes such as synaptic transmission [41]. In fact, ΔN-Bcl-xL produces large conductance ion channel activity in the outer mitochondrial membrane and may induce cell death by contributing to the increase in the conductance of this membrane and the release of cytochrome c [38,39]. Moreover, the accumulation of ΔN-Bcl-xL is associated with mitochondrial injury or neuronal injury in both in vitro and in vivo models of cerebral ischemia [39,42,43,44]: glutamate-mediated excitotoxicity and ischemic strokes enhance the formation of endogenous ΔN-Bcl-xL in hippocampal neurons, promoting neuronal death [31,43]. All these modifications provide diverse roles for Bcl-xL during the lives of the nervous system’s cells [45,46], playing both anti-apoptotic and pro-survival cellular roles [47,48], making Bcl-xL an essential protein in cell fate.

We have previously shown that recombinant Bcl-xL, depleted of its transmembrane segment located at the C-terminal end of the protein (called Bcl-xL-ΔTM) [45,49,50], can undergo various types of aggregation depending on temperature: amyloid-like fibrils are obtained at 37 °C, or amyloid fibrils are formed at 70 °C [51]. *In cellulo,* recombinant Bcl-xL-ΔTM, internalized in a human neuroblastoma cell line, SH-SY5Y, acquires biochemical modifications when the cells are exposed to stress with staurosporine. Bcl-xL-ΔTM accumulates, aggregates, and exhibits amyloid features [51]. In this study, we mimicked the caspase cleavage of the N-terminal fragment of the loop domain (between Arg78Glu79) of Bcl-xL-ΔTM with enzymatic proteolysis by trypsin, and we generated fragments after the incubation of the ΔN-Bcl-xL-ΔTM under physiological conditions, i.e., at neutral pH and at 37 °C. We could monitor the formation of canonical amyloid fibers. We generated conformational and highly specific monoclonal antibodies (mAbs) by mouse immunization against ΔN-Bcl-xL-ΔTM amyloid fibers (further called, in this study, BclxLcf37). Three mAbs were selected and characterized *in cellulo*, for studying the behavior of endogenous Bcl-xL in SH-SY5Y upon apoptotic cell death processes, i.e., those induced by transition-metal oxidative stress (Cu, Fe, and Zn).

Such specific conformational antibodies against Bcl-xL amyloid fibers constitute powerful tools for identifying the different forms of Bcl-xL implicated in the amyloid genesis of the apoptosis process. Regarding new therapies, c-mAbs might constitute specific tools for detecting and targeting Bcl-xL as a strategic protein target in brain injuries relevant to stroke or neurodegenerative diseases.

## 2. Results

### 2.1. Trypsin Proteolysis of Bcl-xL-ΔTM Monomer Induces Its Conversion into Amyloid Fibrils

**Figure 1:**
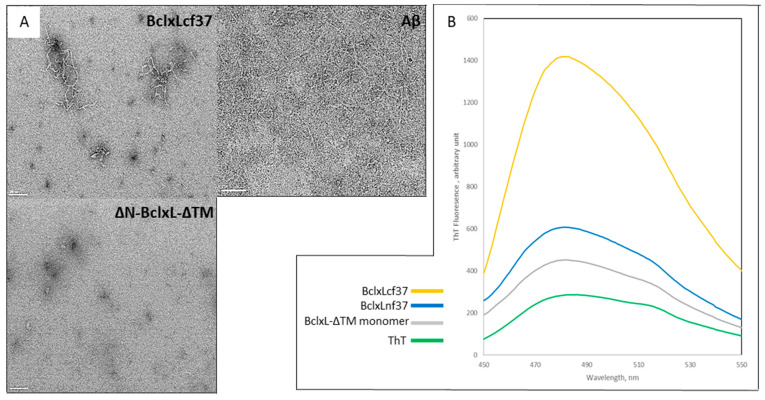
Trypsin-cleaved Bcl-xL forms amyloid fibers. (**A**) Transmission electron microscopy shows fibrils obtained from incubation of the cleaved Bcl-xL-ΔTM form at 37 °C (BclxLcf37); BclxLcf37 fibrils exhibit a similar shape to that of Aβ fibrils, and with a completely different structure from the cleaved Bcl-xL-ΔTM monomer, which appears as an amorphous structure. Scale bar represent 100 µm (**B**) BclxLcf37 fibrils present a high amyloid character as determined by Thioflavin-T fluorescence measurements: yellow curve represents results of fluorescence measurements of BclxLcl37 obtained from incubation of cleaved Bcl-xL-ΔTM at 37 °C; blue curve represents results of fluorescence measurements of native and entire Bcl-xL fibers (BclxLnf37) obtained at 37 °C; gray curve represents results of fluorescence measurements of Bcl-xL-ΔTM monomer; green curve represents results of fluorescence measurements of Thioflavin-T.

Transmission electron microscopy pictures showed that the cleaved Bcl-xL-ΔTM monomer was able to undergo fibril formation when incubated at 37 °C (Figure 1A), and similarly to Aβ. These fibers, labeled BclxLcf37, exhibit the tinctorial characteristics of amyloid fibrils, revealed by measuring ThT dye fluorescence (Figure 1B). After 24 h of incubation, a high difference in intensity was already measured between fibers formed with the entire native Bcl-xL-ΔTM protein and the cleaved protein, ΔN-Bcl-xL-ΔTM, indicating a stronger amyloid feature for the fibers formed with the cleaved protein. Finally, the fluorescence of the Bcl-xL-ΔTM monomer showed a weaker signal than that of BclxLnf37, as described previously [51]. Overall, these results reveal that the formation of fibrils by the ΔN-Bcl-xL-ΔTM is accompanied by profound conformational changes in the native structure, leading to the protein’s aggregation into fibers with canonical amyloid features.

### 2.2. Production, Selection, and Characterization of Conformational Antibodies Directed against BclxLcf37 (c-mAbs)

**Figure 2:**
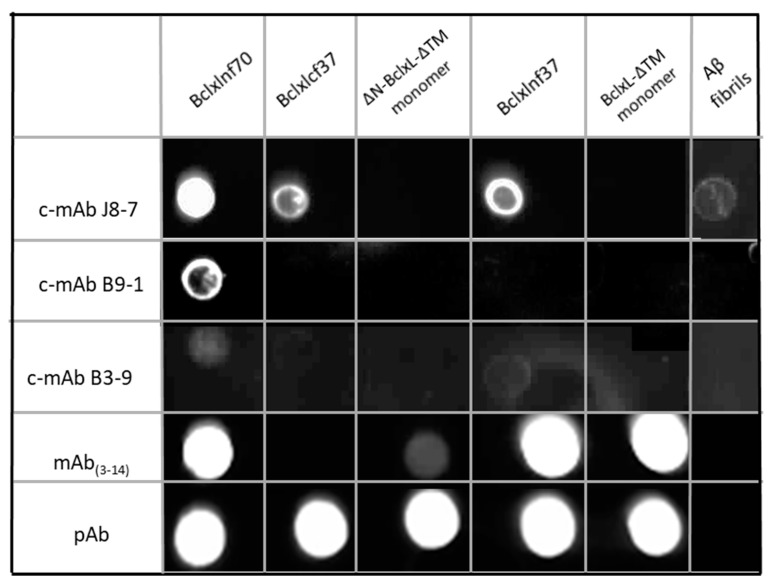
Amyloid specificity determined by dot blots: To test the specificity of c-mAbs J8-7, B9-1, and B3-9′s immuno-recognition, 10 µg of BclxLnf70, BclxLnf37, ΔN-BclxL-ΔTM monomer, BclxLcf37, BclxL-ΔTM monomer, and Aβ fibers were spotted for dot blot immuno-detection. Controls were realized using commercial monoclonal (mAb(3–14)) and polyclonal (pAb) anti-Bcl-xL antibodies.

Following immunized mouse ascites retrieval, selection by ELISA tests (Supplementary Table S1), and the fusion of hybridomas, three conformational monoclonal antibodies (c-mAbs) were isolated because of their specificity in recognizing only Bcl-xL in an amyloid conformation: c-mAb J8-7, c-mAb B9-1, and c-mAb B3-9. Their specificity was tested using dot blot experiments with the three types of Bcl-xL fibers: Bclxlnf37 (low amyloid features), Bclxlnf70 (high amyloid features), and Bclxlcf37 (high amyloid features) (Figure 2 and Supplementary Table S2). Bcl-xL-fiber-specific recognition was addressed by comparing the signals of the c-mAbs J8-7, B3-9, and B9-1 with those obtained with commercial polyclonal and monoclonal antibodies. The J8-7 antibody recognized the three types of Bcl-xL fibers, with a stronger affinity for BclxLnf70 and a medium one for BclxLnf37 and BclxLcf37, and it did not recognize either monomeric Bcl-xL-ΔTM or ΔN-Bcl-xL-ΔTM. By contrast, the B9-1 antibody recognized only BclxLnf70 with a medium affinity. The third c-mAb, B3-9, recognized, with a weak affinity, both fibers generated with native Bcl-xL. Among the three c-mAbs, only J8-7 also recognized, in a weaker fashion, Aβ amyloid fibers. Thus, we generated three different conformational mAbs with different specificities: J8-7 recognizes the three Bcl-xL fibers and, particularly, the amyloid form, whereas B9-1 recognizes, more specifically, the amyloid form of native Bcl-xL fibers, and B3-9 recognizes native Bcl-xL fibers.

As expected, commercially available polyclonal antibodies (pAb) recognized every form of Bcl-xL (native and cleaved monomer, and the three Bcl-xL fibers), whereas a commercial monoclonal antibody targeting the peptide encoding the sequence 3–14 of Bcl-xL (mAb_(3–14)_) recognized only the full form of Bcl-xL-ΔTM (monomer and Bclxlnf37). Commercial Abs were unable to recognize Aβ fibers. It may be noted that mAb_(3–14)_ bound to ΔN-Bcl-xL-ΔTM monomer very weakly and not to the cleaved Bcl-xL fiber, BclxLcf37. This could be due to the fact that the cleaved monomer solution used for the dot blot, ΔN-Bcl-xL-ΔTM, contained all the fragments generated by the enzymatic trypsin cleavage. However, Bclxlcf37 was not recognized by mAb_(3–14)_, suggesting the absence of the N-terminus in the fiber.

### 2.3. SH-SY5Y Cells as a Model to Monitor the Apoptosis Induced by Metal Stress

The oxidative conditions induced by metal stress were confirmed by measuring the kinetics of the reactive oxidative species (ROS) production during the 24 h (Supplementary Figure S2). Copper led to a high and rapid response (88% compared to positive control, at 100%, with H_2_O_2_) within the first 5 h, which thereafter slowed down to 66% within 24 h, whereas iron and zinc induced lower amounts of ROS, which reached 7% and 25%, respectively, within 24 h.

**Figure 3:**
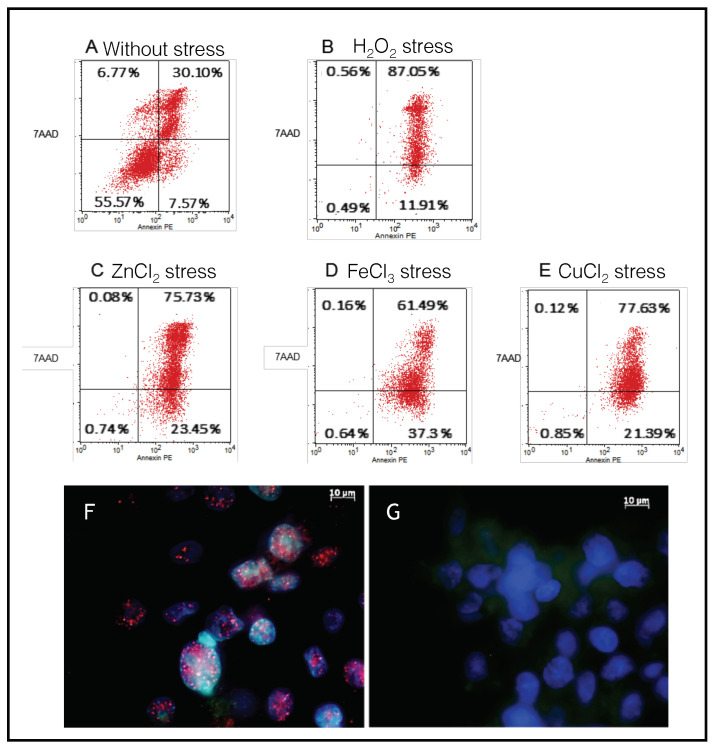
Metal stress-induced SH-SY5Y apoptosis. Annexin V-PE and 7AAD staining assay. Control experiments consisted of negative controls without stress (**A**) and positive controls with H_2_O_2_ stress (100 µM) (**B**). FACS analysis of Annexin V-PE and 7AAD fluorescence signals in SH-SY5Y treated with 150 µM ZnCl_2_ (**C**), 300 µM FeCl_3_ (**D**), or 500 µM CuCl_2_ (**E**) for 24 h. Metal concentrations were selected based on the concentration-dependent death induced (see Supplementary Figure S1). Cells totally unlabeled represent alive cells; cells simply labeled with Annexin V-PE represent cells engaged in the early apoptosis phase; cells simply labeled with 7AAD represent cells engaged in the necrotic phase; cells double-labeled with Annexin V-PE and 7AAD represent cells engaged in late apoptotic and necrotic processes. Caspase-9 activation (**F**–**G**). (**F**) Caspase-9 immunostaining following FeCl_3_ (300 µM) apoptotic stress induction (Alexa-594/caspase-9 antibody immunostaining); (**G**) SH-SY5Y without metal stress.

Cell death induced by iron, copper, and zinc was analyzed by flow cytometry assays using double staining with annexin V-PE and 7AAD antibodies (Figure 3). The results, expressed as percentages of cells labeled by annexin V-PE and/or 7AAD, show that iron, copper, and zinc stress, applied for 24 h to SH-SY5Y cells, mainly induced apoptosis: 23% of the cells for zinc (Figure 3C), 37.3% of the cells for iron (Figure 3D), and 21% of the cells for copper (Figure 3E) were in the early phase, and 75% of the cells for zinc, 61% of the cells for iron, and 78% of the cells for copper were already in the late apoptotic phase. H_2_O_2_ treatment (Figure 3B) constituted an efficient positive control to induce apoptosis at the end of cells’ incubation: 87% of cells reached the late phase of apoptosis, and 12% were still in the early phase.

To support the characterization of the cell death process induced by metals, immunostaining with an antibody, anti-caspase-9, was carried out on cells incubated with (Figure 3F) or without metals (Figure 3G). Confocal images of the cells incubated with iron showed significant increases in red fluorescent staining (red dots) compared to in control cells, confirming caspase cascade activation related to apoptotic cell death. Apoptosis was also revealed by the appearance of nucleus bodies (blue spots, Hoechst staining), a classic morphological DNA fragmentation step representative of programmed cell death. Similar caspase-9 activation was observed in cells incubated with copper and zinc.

### 2.4. Endogenous Bcl-xL Is Converted into Amyloid Aggregates Following Apoptosis Induced by Metal Stress in SH-SY5Y Cells

**Figure 4:**
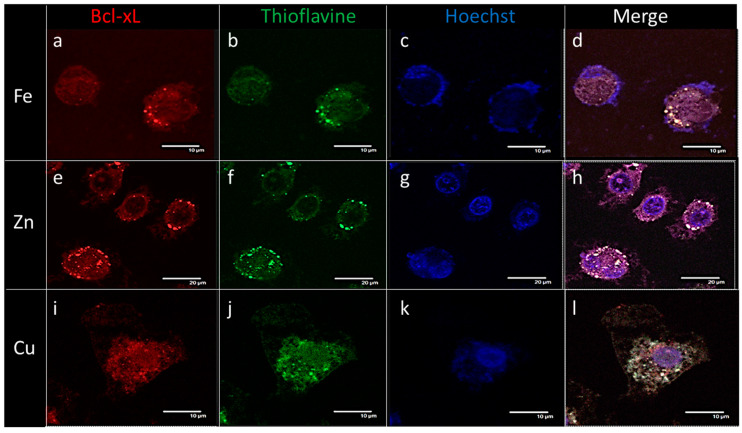
Metal stress-induced intracellular Bcl-xL amyloid deposits in SH-SY5Y: following iron (**a**–**d**), zinc (**e**–**h**), and copper (**i**–**l**) stress, immunostaining with commercial polyclonal anti-Bcl-xL antibodies and Thioflavin-T. Small white arrows indicate the same Bcl-xL aggregates recognized by the monoclonal antibody (**a**,**e**,**i**; visualized at 594 nm, red color) and stained with the amyloid dye (**b**,**f**,**j**; visualized at 488 nm, green color). Hoechst dye stained the nucleus (**c**,**g**,**k**; visualized at 405 nm, blue color). Pictures (**d**,**h**,**l**) resulted from the fluorescence merging of the three stains and reveal the co-localization of Bcl-xL aggregates with Thioflavin-T dye (white spots).

**Figure 5:**
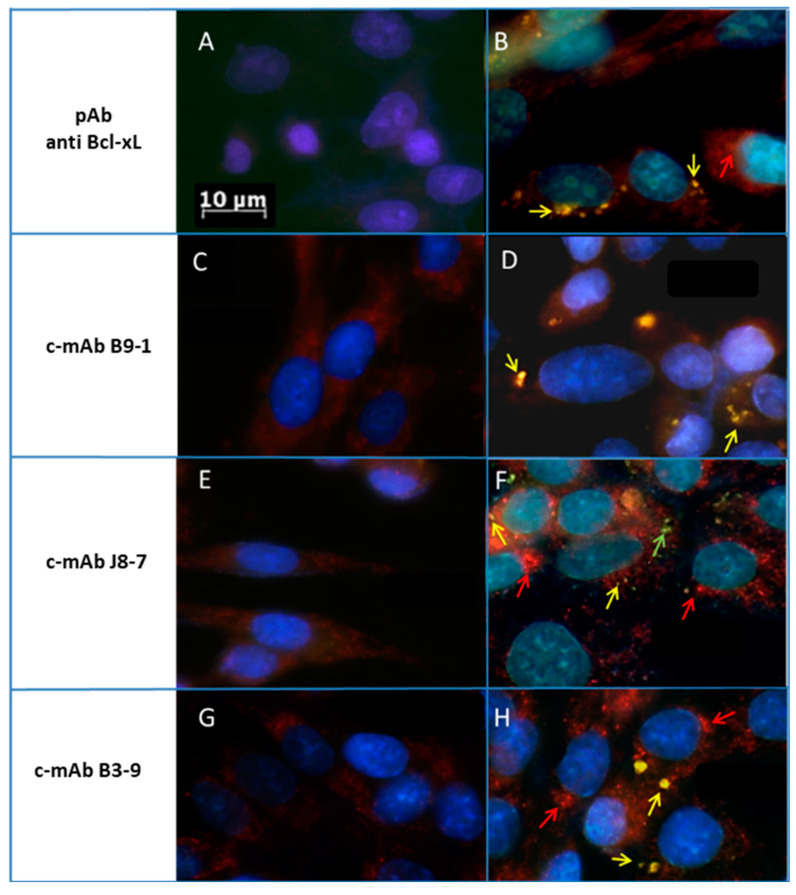
Cellular Bcl-xL detection by immunohistochemistry in SH-SY5Y culture chemically stressed with iron, using commercial polyclonal Bcl-xL antibodies (**A**–**B**) and c-mAb (**C**–**H**) (visualized at 594 nm, red color) and Thioflavin-T (visualized at 488 nm, green color). Hoechst dye stained the nucleus (visualized at 405 nm, blue color). Pictures resulted from the merging of the three stains. Amyloid Bcl-xL aggregate appeared in yellow as indicated by the arrows. We can note that some aggregates appear in green, suggesting other protein types of amyloid deposits.

The formation of amyloid fibrils by endogenous Bcl-xL was observed in metal-stressed SH-SY5Y cells. Cellular immunohistochemistry performed 24 h after metal stress (Figure 4) showed intracellular Bcl-xL whose specific antibody staining co-localized with thioflavin staining, used as a dye to identify amyloid fibrils. Immunochemistry pictures of cellular cultures incubated with different metals showed Bcl-xL, represented by red fluorescence, imaged at λ = 594 nm (Figure 4a,e,i), and ThT green fluorescent signals, imaged at λ = 488 nm (Figure 4b,f,j), which also co-localized within Bcl-xL deposits (Figure 4d,h,l, white arrows). Altogether, these results indicated that during the oxidative apoptotic process, cellular Bcl-xL underwent morphological changes, which led to its accumulation in amyloid aggregates.

c-mAbs allow the immuno-detection of intracellular Bcl-xL amyloid aggregates following apoptosis induced by metal stress in SH-SY5Y cells *:* we then investigated if c-mAbs were suitable for both detecting Bcl-xL aggregates and characterizing these aggregates, in neuroblastoma cells under iron stress (Figure 5). In the control condition, i.e., without stress, we observed a weak and diffuse staining that was likely to be non-specific (Figure 5A,C,E,G). In the stressed condition, all the c-mAbs, B3-9, J8-7, and B9-1, detected aggregates, and their signals appeared in yellow, indicating an overlap between the two types of staining (yellow arrows), both ThT (green color, visualized at 488 nm) and c-mAbs (red color, visualized at 594 nm), indicating the presence of Bcl-xL in an amyloid conformation, as we described previously with the metal stress assay (Figure 4). For B9-1 (Figure 5D), the Ab recognizing the amyloid fibers of Bcl-xL only composed of the uncleaved protein, BclxLnf70 (Supplementary Table S2), we found a perfect overlap between the signals of immunostaining and ThT (yellow arrows). J8-7 and B3-9 (Figure 5F,H) recognized every type of Bcl-xL aggregate (Supplementary Table S2), and whereas some overlapping signals were still observed, some of the spots labeled with c-mAbs were not stained by ThT (red arrows). Therefore, these c-mAbs allowed the detection of coexisting amyloid and non-amyloid Bcl-xL aggregates within cell cultures. Moreover, association with ThT staining enabled discrimination between the two types of aggregates. B9-1 allowed probing the amyloid character of Bcl-xL co-localizing with ThT staining and, further, the conclusion that Bcl-xL was present within the amyloid deposits in an amyloid conformation and not simply as a native protein. Interestingly, we also observed in the SH-SY5Y cultures some aggregates stained with ThT (green arrow), indicating that these aggregates were amyloid but constituted by other types of protein, supporting the specificity of c-mAbs against Bcl-xL.

### 2.5. The B3-9 c-mAb Specifically Immunoprecipitates Bcl-xL from Cells Subjected to Metal-Induced Apoptosis

**Figure 6:**
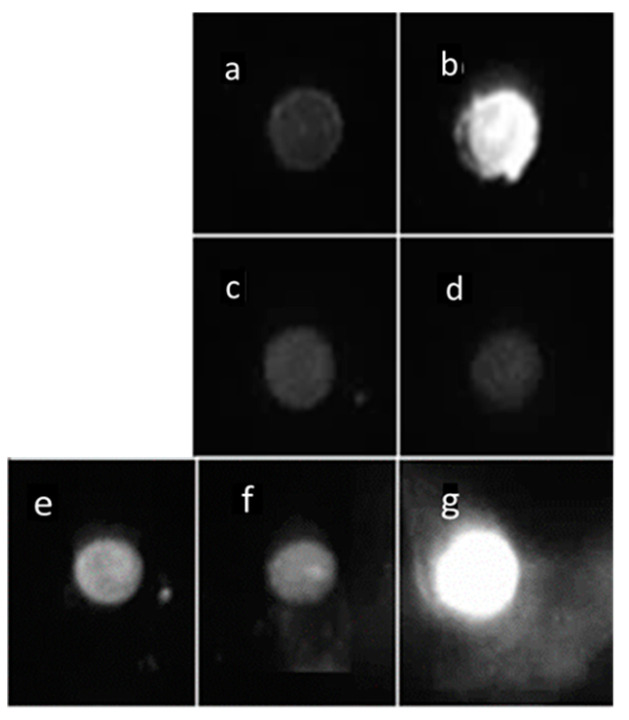
Dot blot of immunoprecipitation (IP) pull-down using B3-9 c-mAb: IP of cell fraction obtained after centrifugation and revealed with the polyclonal anti-Bcl-xL antibody: (**a**,**c**) fraction obtained from SH-SY5Y control and (**b**,**d**) SH-SY5Y stressed for 24 h with FeCl_3_; (**a**,**b**) resulted from IP pull-down; (**c**,**d**) resulted from the flow-through of the IP and constituted the negative control. (**e**–**g**) constituted the positive controls with blotting for (**e**) BclxLcl37, (**f**) BclxLnf70, and (**g**) BclxL-ΔTM monomer.

The capacity to immunoprecipitate Bcl-xL amyloid fibers was tested with the three c-mAbs, B3-9, J8-7, and B9-1. Only B3-9 pulled down Bcl-xL from iron-stressed cells (Figure 6b). Dot blot assays were stained using a commercial polyclonal anti-Bcl-xL. The recognition of BclxLcf37, BclxLnf70, and Bcl-xL-ΔTM monomers by the antibody confirmed its specific recognition of the proteins of interest (Figure 6e–g). The absence of the recognition of proteins in the flow-through fraction confirmed the efficiency of B3-7 when used for IP experiments (Figure 6c,d).

## 3. Discussion

The cell’s pro-survival Bcl-xL, a member of the Bcl-2 family, can form, in vitro, two forms of native-like fibers depending on the temperature of its incubation [51]. Like several other members of the Bcl-2 family, Bcl-xL can be cleaved by caspases, enhancing the pro-death activities of these proteins in apoptosis [52]. In this study, firstly in vitro, we show that Bcl-xL, upon caspase like-cleavage, is able to undergo structural modification and aggregation into amyloid fibers. The N-terminal sequencing of Bcl-xL fragments shows that trypsin exhibits a specific proteolytic activity, located within the Arg78Glu79 regulation loop of Bcl-xL, very close to the caspase-3 cleavage site in the loop domain (between Asp76 and Ala77), releasing the BH4-domain associated with an α-helix [17,34,53]. Bcl-xL cleavage has also been reported to result from different stimuli such as IL-3 withdrawal, virus infection [38], caspase-3-like proteases from an apoptotic CTLL-2 cell lysate [33], or the direct activation of caspase-9 by chemically induced dimerization [54]. The endogenous cleavage of Bcl-xL by caspases or calpains produces a C-terminal protein (ΔN-Bcl-xL) with a Bax-like pro-apoptotic activity [38,39]. To our knowledge, this is the first report that shows in vitro, such cleaved Bcl-xL proteins, ΔN-BCL-xL-ΔTM, at a physiological temperature of 37 °C, form fibers with amyloid tinctorial characteristics, as revealed by ThT fluorescence. Further characterization of BclxLcf37 will help to define more of its biophysical features.

To study amyloid BclxLcf37 fibers, and to know whether they exist in vivo and in which cellular context they are found, specific monoclonal antibodies (mAbs) have been produced, by mouse immunization, against the different conformational epitopes of Bcl-xL. Three antibodies (c-mAbs) were selected for their capacity to recognize, specifically, Bcl-xL amyloid fibers [51,55]. Antibody property analysis indicates that all the c-mAbs recognized amyloid or amyloid-like fibers and did not interact with the monomeric states of either native or cleaved Bcl-xL, in contrast to commercial monoclonal and polyclonal antibodies. We can note that commercial mAbs do not recognize Bclxlcf37, and this may be also due to the loss of its BH4 domain following the cleavage.

In order to explore its biological role, we examined endogenous Bcl-xL’s fate in neural cells under metal-induced oxidative stress, as it is highly expressed in the human brain and could play an important role in neurodegenerative diseases [40].

Transition metals, causing oxidative stress, can induce apoptosis in neural cells as well as neuroinflammation; both are associated with neurodegenerative diseases [56]. In human neuroblastoma (SH-SY5Y), oxidative apoptosis was induced after 24 h of exposure to metallic stress (FeCl3, ZnCl2, and CuCl2) which produce ROS. This phenomenon is associated with the activation of the caspase-9, which indicates the activation of the mitochondrial apoptotic pathway together with the involvement of the Bcl-2 family of proteins. After 24 h of incubation, according to fluorescence microscopy, Bcl-xL was immuno-localized in intracellular aggregates with amyloid features, as revealed by ThT staining. Overall, these results indicate that the disruption of metal homeostasis provokes cell apoptosis and leads to conformational changes in Bcl-xL.

Metal imbalance, even at low concentrations, could readily foster the oligomerization and aggregation of several proteins implicated in neurodegenerative diseases [3]. Thereby, Aβ oligomerizes with Cu and Zn [57], tau protein oligomerizes in presence of the trivalent metal ions Al(III) and Fe(III) [58], and αSyn can also oligomerize in the presence of different metals including Al(III), Cu(II), Cd(II), and Fe(III) [59]. This enhancement, readily favored by the presence of binding sites for neurometals within proteins, leads to metal sequestration into amyloid plaques. The presence of binding sites inside the protein’s structure fosters the aggregation and toxic reactions through an increase in the reactive oxygen species (ROS) concentration through Fenton and Haber–Weiss reactions [60]. The direct consequence of these metal bonds with proteins is a high concentration of sequestered metals in amyloid deposits, which could locally participate in the oxidative stress at the origin of neural apoptosis and of neuronal and synaptic disruption due to the production of ROS. The potential presence of metal binding sites in Bcl-xL remains to be unveiled to understand the pathophysiological significance of amyloid aggregates of Bcl-xL during apoptosis induced by oxidative stress from metals. The new c-mAbs will be useful for deciphering this process and especially since it is described that Bcl-xL is cleaved by caspase-3 inside exosomes in order to participate in cell-to-tumor cell communication [61].

Taken together, these results are of high interest for the in vivo characterization of Bcl-xL behavior in the context of cells undergoing metallic stresses. We chose to overexpose neuroblastoma cells to metals essential for life, i.e., Fe, Cu, and Zn, which are found in high concentrations in the brains of patients with neurodegenerative diseases. We confirmed that metal-induced apoptosis is associated with ROS production and caspase-9 activation and leads to endogenous Bcl-xL accumulation in intracellular and amyloidogenic aggregates. Using the c-mAb specific for Bcl-xL fibers, we have shown that Bcl-xL is included in aggregates of amyloid structures after apoptosis induction. Moreover, one of these c-mAbs can be used to isolate Bcl-xL fibers for further structural analyses. These c-mAbs strongly recognize Bcl-xL aggregated in amyloid fibers: the c-mAbs co-localizes only with the amyloid dye Thioflavin T in cells under apoptotic stress.

In conclusion, we show that the Bcl-xL protein can undergo dramatic conformational changes both *in cellulo* and in vitro under oxidative conditions, leading to the accumulation of Bcl-xL in aggregates of amyloid features. The self-propagating behavior of Bcl-xL appears to be strongly dependent on its biophysical state, as described for the canonical amyloid aggregation of the amyloid protein, i.e., Aβ or αSyn [62]. This is supported by the fact that fibrils constituted by the cleaved form of Bcl-xL present the characteristics of canonical amyloidal structures, whereas the fibrils from the native Bcl-xL exhibit native-like conformations [51]. This strongly suggests that Bcl-xL conformational changes result from caspase activation in apoptotic cells. The conformational antibodies characterized in this study will allow the identification of the structure of Bcl-xL in these aggregates and their protein composition, which might be crucial for understanding their role in the context of neurodegenerative disease initiation and propagation. Finally, these conformational mAbs could also be considered as useful tools for the early diagnosis and the follow-up of brain lesions, using imaging detection in the context of abnormal rates of apoptosis in a neurodegenerative environment.

## 4. Materials and Methods

### 4.1. Generation of Fibrils

The recombinant native Bcl-xL-ΔTM protein used in these experiments was obtained by purification from bacterial production as previously described [51]. Two kinds of Bcl-xL-ΔTM fibrils were generated with the entire native protein, denominated “BclxLnf37” and “BclxLnf70”, depending on the temperature of incubation as previously established in the laboratory [51]. Briefly, BclxLnf37 was formed using a 200 µM concentration of recombinant native Bcl-xL-ΔTM monomer incubated at 37 °C, and BclxLnf70 was obtained with a 100 µM concentration of recombinant native Bcl-xL-ΔTM monomer at 70 °C; both were generated in phosphate buffer (20 mM phosphate buffer, 50 mM NaCl, pH 7, 200 µM AEBSF protease inhibitor (Sigma-Aldrich #A8456)). A new, third type of Bcl-xL fibril, called “BclxLcf37”, was obtained after the cleavage of recombinant native Bcl-xL-ΔTM monomer (200 µM) by enzymatic treatment using trypsin (200 nM, TPCK-treated, Sigma-Aldrich #4352157) at 37 °C for 30 min; the reaction was stopped by the addition of 200 µM AEBSF protease inhibitor. This cleaved protein, ΔN-Bcl-xL-ΔTM, was incubated at 37 °C in phosphate buffer (20 mM phosphate buffer, 50 mM NaCl, pH 7) to generate BclxLcf37 fibrils.

### 4.2. Electron Microscopy

The negative stain Mica-carbon flotation technique (MFT) was used. Samples were adsorbed on the clean side of a carbon film on mica, stained, and transferred to a 400-mesh copper grid. Images were taken under low dose conditions (<10 e^−^/Å^2^) with defocus values between 1.2 and 2.5 μm on a Tecnai 12 LaB6 electron microscope at a 120 kV accelerating voltage using a CCD Camera, Gatan Orius 1000.

### 4.3. Fluorescence Spectroscopy Experiments

Bcl-xL amyloid fibrils were analyzed using a Fluorolog FL3-22 spectrophotometer from Horiba-Jobin Yvon Spex. Fluorescence measurement studies of cleaved or entire Bcl-xL fibrils were carried out using 20 µM protein in the presence of 20 µM thioflavin T analyzed in a Quartz Suprazil cell, at the steady state of the binding of ThT, for fluorescence using a Jasco FP-750 spectrometer (fluorescence: Ex.: 440 nm, Em.: 450–550 nm).

### 4.4. Production and Purification of Specific Conformational Monoclonal Antibodies (mAbs) Against Amyloid Bcl-xL Fibers

Biozzi mice were immunized subcutaneously with 10 mg of native Bcl-xL-ΔTM monomer, BclxLnf70, BclxLnf37, or BclxLcf37 with complete Freund’s adjuvant. Subsequently, 4 to 6 booster injections of 10 mg of the different conformations of Bcl-xL with incomplete Freund’s adjuvant were given subcutaneously at 1-day intervals. After the last injection, the mice were bled and the polyclonal response was tested by ELISA. The mouse that showed the best response against the immunogen was sacrificed for splenic cell fusion according to Kohler and Milstein [63]. Supernatants were screened by ELISA for antibody production (supplementary results, supplementary Table S1). The specificity of the positive supernatants was then tested with different forms of Bcl-xL. Selected hybridoma cells were then cloned by the limiting dilution technique, tested by ELISA. Positive clones were cultivated for ascites production. Four fusions with mice injected with different forms of Bcl-xL were performed. Monoclonal antibodies were purified from ascites by precipitation with ammonium sulfate, centrifuged, and dialyzed against PBS. Through this process, three mAbs were selected for their efficiency in targeting Bcl-xL-positive fibers/oligomers and, at the same time, not recognizing Bcl-xL-ΔTM monomer. These mAbs were produced from hybridomas called B9-1, B7-3, and J8-7. In the rest of the article, these names will be used as the mAb names. The cell lines producing the antibodies described in this article are available on request from the Collection Nationale de Cultures de Microorganismes (CNCM) at Institut Pasteur, Paris: https://www.pasteur.fr/fr/recherche-cncm. This study was approved by Minister of French Research and Institut Pasteur ethics committee (project n° 00802.02, 20/11/2015).

### 4.5. Dot Blotting

Conformational antibody characterization was performed by dot blotting using BclxLnf37, BclxLnf70, BclxLcf37, Bcl-xL-ΔTM monomer, Aβ fibers and Aβ monomer as a target on nitrocellulose membranes (Hybond-ECL—Amersham Biosciences). Following a short centrifugation, 10 µg of BclxLnf70, BclxLnf37, BclxLcf37, or Aβ fibrils and of Bcl-xL-ΔTM monomer were spotted on the membrane and dried for 30 min. After blockage at room temperature with PBS-Tween 20/10% milk and washing in PBS-Tween 20/5% milk, the membranes were incubated with either a rabbit polyclonal anti-Bcl-xL Ab (BD Bioscience; 1/2000) or mouse monoclonal anti-Bcl-xL Ab _(3–14)_ (obtained against the N-terminal part (3–14) of Bcl-xL; BD Bioscience; 1/2000) or with the mAbs to be characterized (1/5000) in PBS-Tween 20/5% milk. After three washes, they were incubated with a secondary goat anti-rabbit or rabbit anti-mouse Ab coupled to HRP (horseradish peroxidase) (BETHYL laboratories; 1/5000) in PBS-Tween 20/5% milk. Binding was revealed with a mixture of the SuperSignal^TM^ West Pico PLUS Chemiluminescent Substrate (ThermoScientific #34579). Signals were observed using the Molecular Imager device (Fusion FX7, ThermoFischer).

### 4.6. Cell Culture

Human SH-SY5Y neuroblastoma cells (ATCC #CRL 2266) were grown in Dulbecco’s modified Eagle’s medium F-12 (DMEM: F-12) supplemented with 1% penicillin–streptomycin and 10% fetal calf serum (Invitrogen). Adherent cells were grown in DMEM/F12 1:1 (Dulbecco’s modified Eagle’s medium) supplemented with 2 mM L-glutamine and 1.2 g/L sodium bicarbonate, 1% penicillin–streptomycin (Invitrogen), and 10% heat-inactivated fetal calf serum (Invitrogen), at 37 °C, under 5% CO_2_.

### 4.7. Metal Oxidative Stress

Iron (FeCl_3_, Sigma, France), copper (CuCl_2_, Acros Organics, France), and zinc (ZnCl_2_, Acros Organics, France) were used to induce oxidative stress in SH-SY5Y cells. Metal solutions were added to the culture medium at the following concentrations: [FeCl_3_] = 300 µM, [CuCl_2_] = 500 µM, and [ZnCl_2_] = 150 µM. These metal concentrations correspond to the effective concentrations causing the cell death of 50% of the cell population after 24 h of incubation (Supplementary Figure S1). Hydrogen peroxide at 100 µM (H_2_O_2_, Sigma #216763) was used as a positive control for oxidative stress and apoptosis. Unstressed cells were used as negative controls. The metal solutions were applied for 24 h, whereas the stress by hydrogen peroxide was applied for 30 min, and then, the medium was replaced with fresh medium and the cells were incubated for 24 h.

### 4.8. Flow Cytometry

Apoptosis was evaluated using an Annexin V-PE Apoptosis Detection Kit (BD Pharmingen). Cells were recovered after 24 h of stress and were suspended in 1X binding buffer solution to obtain 10^6^ cells/mL. We collected 100 µL of cells and stained them with 5 µL of annexin V labeled with phycoerythrin (PE) and 5 µL of 7-amino-actinomycin (7AAD). After 15 min of incubation, we performed the measurement of the signal emitted by labeled cells on a FACS Calibur flow cytometer (Becton Dickinson, Franklin Lakes, NJ, USA). The specific signal was adjusted according to controls consisting of 1) unstressed cells and unlabeled cells, 2) cells stained only with annexin V-PE, and 3) cells stained only with 7AAD.

### 4.9. Immunofluorescence

Cells were seeded and cultivated on poly-ornithine-coated glass 24 h before the application of metal stress. Following metal treatments, cells were fixed with 5% formalin for 20 min at room temperature, after 3 washes of 5 min, and then permeabilized with 80% methanol at −20 °C for 6 min, followed by 1 h 30 min of saturation with PBS-NP-40 at 0.05%. The immunostaining was performed with primary antibodies in PBS-NP40 0.05%: mouse monoclonal and rabbit polyclonal anti-Bcl-xL antibodies (BD Biosciences; 1/1000), and rabbit anti-caspase-9 antibody (USBiological; 1/1000). The primary antibodies were incubated overnight at 4 °C, and following 3 washes, the secondary rabbit anti-mouse antibodies coupled with Alexa-546 or goat anti-rabbit antibodies coupled with Alexa-647 or Alexa-594 (Invitrogen; 1/1000) were incubated for 1 h, at room temperature, in PBS-NP-40 at 0.05%. Then, Thioflavin T (Sigma-Aldrich, #T3516) at 0.05% in H_2_O was used to specifically stain amyloid aggregates (5 min at 4 °C in the dark). The coated pieces of glass were mounted on slides with mounting medium containing Hoechst dye, DABCO (1,4-diazabicyclo [2,2,2] octane, Sigma), and Mowiol. The slices were observed for Alexa-546 (λexc = 543 nm; λem = 590 nm), Alexa-647 (λexc = 637 nm; λem = 650 nm), Hoechst (λexc = 330 nm; λem = 450 nm), and Thioflavin T (λexc = 488 nm; λem = 515 nm) staining using a confocal microscope (Leica TCS-SP2).

### 4.10. Immuno-Precipitation (IP) Experiments

Oxidative stress was applied to confluent cells 24 h before lysis. The supernatants were removed from the cells, which were put on ice with 2 mL of RIPA lysis buffer (NaCl 150 mM, 1% NP-40 lysis buffer; 0.4% deoxycholate; Tris, 50 mM; pH 7.4) + 1X Halt Protease Inhibitor Cocktail (#RH238656, ThermoFisher) for 30 min. Lysed cells were suspended mechanically using scrappers and added back to their respective supernatants. A first centrifugation with a Centrifuge 5810 R (Eppendorf) at 1000× *g* for 10 min helped to remove nuclei and whole cells; the pellet was discarded. A second at 10,000× *g* for 20 min helped to remove microsomes, small vesicles, ribosomes, macromolecules, and soluble proteins. An ultracentrifugation at 80,000× *g* for 1 h with an Optima XPN Ultracentrifuge (Beckman Coulter) enabled the retrieving of non-soluble proteins in the pellet, which were used for further experiments. Immunoprecipitation was performed using Protein G coated with sepharose (Protein G Sepharose^TM^ 4 Fast Flow #17-6002-35, GE Healthcare) washed with lysis buffer. Pre-clearing for 1 h with a 50% bead suspension was systematically carried out before adding antibodies at their recommended concentrations. The incubation with antibodies was performed for at least 2 h at 4 °C under rotation, and 100 µL of Protein G beads were then added per 500 µL of aliquots to allow the formation of immune complexes. The flow-through was retrieved, and complexes were dissociated owing to successive suspensions in lysis buffer, basic buffer (NaH_2_PO_4_ 0.2 M, NaCl 0.13 M, pH 8) and acidic buffer (Na_2_HPO_4_ 0.2 M, citric acid 0.1 M, pH 3), with a centrifugation between each step to remove the supernatant. Binding was revealed by dot blotting with a commercial polyclonal anti-Bcl-xL (pAb) (Invitrogen) on the nitrocellulose membranes where the samples were deposited.

## Supplementary Materials

Supplementary materials can be found at https://www.mdpi.com/1422-0067/21/20/7625/s1.
