# Supplementary material for: Development of Conformational Antibodies to Detect Bcl-xL’s Amyloid Aggregates in Metal-Induced Apoptotic Neuroblastoma Cells
Source: Int J Mol Sci. 2020 Oct 15;21(20):7625. doi: 10.3390/ijms21207625 (PMC7589975; doi:10.3390/ijms21207625)
Supplement: Supplementary file 1 [file ijms-21-07625-s001.pdf]

Supplementary Materials

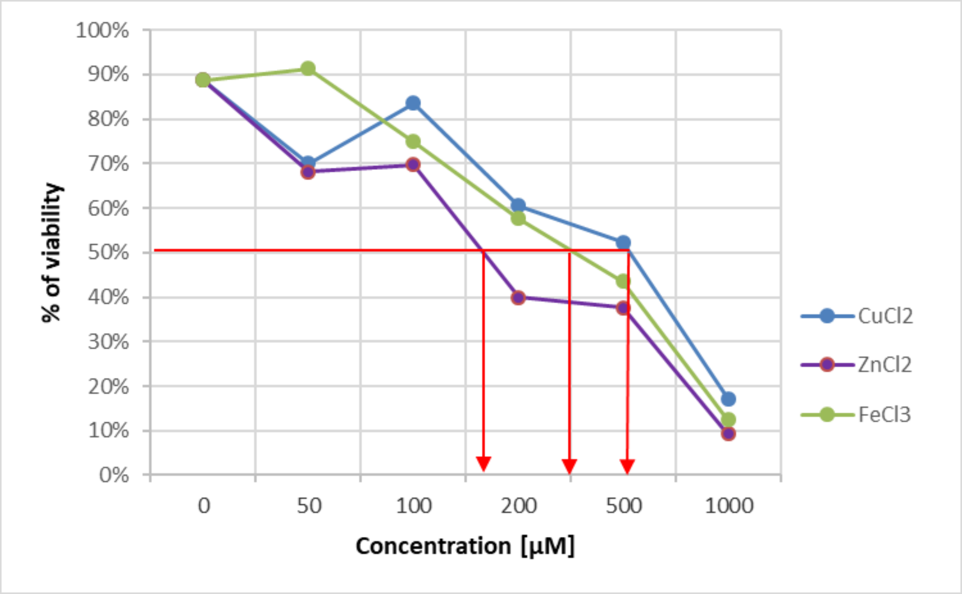

Supplementary Figure S1. Determination of working metal concentration: The determination of concentrations effective for obtaining 50% cell viability was performed at the end of 24 h of incubation of SH-SY5Y with metals $CuCl_2$, $ZnCl_2$, and $FeCl_3$ at different concentrations. The 50% effective concentrations were evaluated by direct counting under a light microscope (Olympus CK41) using a Malassez chamber. Briefly, after detachment of cells by trypsinization, 10 µL of cell suspension was mixed with 10 µL of trypan blue. Ten microliters of this mixture was put into the Malassez chamber for counting. Viability was checked by distinguishing viable (white/transparent cells) from dead (blue) cells, in which the membranes are damaged and allow the entry of trypan blue dye into the cells. Then, results were plotted and $EC_{50\%}$ were deduced as follows: [$ZnCl_2$] = 150 µM ; [$FeCl_3$] = 300 µM; [$CuCl_2$] = 500 µM.

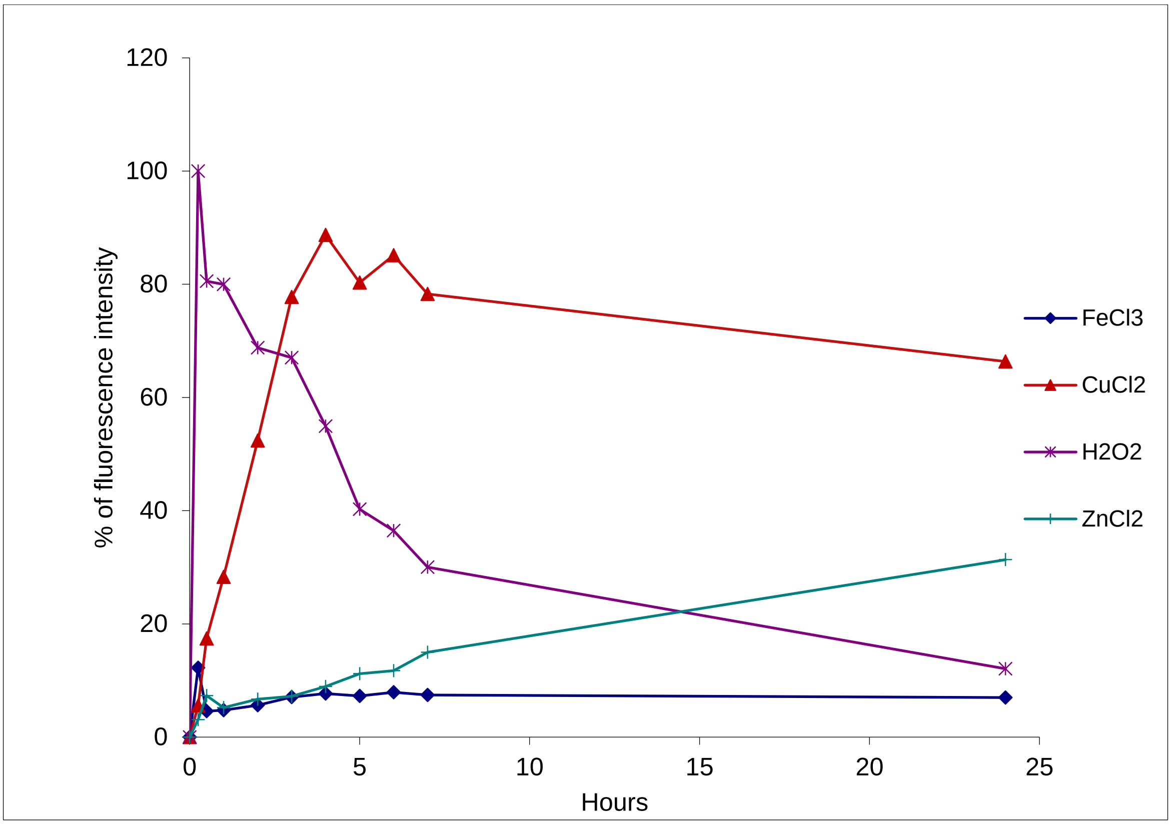

Supplementary Figure S2. Metal stress-induced reactive oxygen species production. Intracellular ROS accumulation was measured by fluorescent intensity with a plaque spectrophotometer (Tecan Infinite M200) using a ROS detection reagent (Molecular Probes #C400). SH-SY5Y cells were seeded into a 48-well plate (100,000 cells/well) and incubated for 24 h with culture medium without red phenol. Then, metal stress was applied to study the kinetics for 24 h. At several time points, one well with each metal was washed and replenished with culture medium containing carboxy-H2DCFDA fluorescent dye (2′7′-dichlorodihydrofluorescein diacetate) at 10 µM for 1 h at 37 °C in the dark. At the end, the dye was removed and replaced with culture medium without red phenol, and the fluorescence signals of each well were subsequently detected and measured using an excitation wavelength of 495 nm and emission wavelength of 525 nm. Results are expressed as the fluorescence percentages compared to the positive controls (H_2O_2, representative of 100%) and the negative controls (untreated cells, representative of 0%).

Supplementary Table S1. Monoclonal conformational antibody production. Biozzi immunized mice were bled after the last injection, and the polyclonal response was tested by ELISA. The mouse that showed the best response against the immunogen was sacrificed for splenic cell fusion according to Köhler and Milstein [1]. Supernatants were screened by ELISA for antibody production. The specificity of positive supernatants was then tested with different forms of Bcl-xL. Selected hybridoma cells were then cloned by the limiting dilution technique, tested by ELISA. Positive clones were cultivated for ascites production. Three fusions with mice injected with different forms of Bcl-xL were performed. Monoclonal antibodies were purified from ascites by precipitation with ammonium sulfate, centrifuged, and dialyzed against PBS.

Hybridomas Tested Against	cl-Fb 37	cl-Fb 37	Nat-Fb 37 ou 70	Monomer
**J8-7**	1.985	1.654	2.151	2.001
**B7-3**	3.13	2.968	3.059	2.956
**B9-1**	2.72	2.789	3.171	2.909

Supplementary Table S2. Recapitulation of results with the intensity of Bcl-xL recognition and the values of obtained results as a function of the total number of assays of Bcl-xL immuno-detection by dot blots with the different c-mAbs.

Blotted Samples / Antibodies	BclxLnf70	BclxLcf37	ΔN-BclxL-ΔTM Monomer	Bclnf37	BclxL-ΔTM Monomer	Aβ Fib
c-mAb J8-7	+++<br>3+/3	++<br>4+/4	-<br>2-/2	++<br>4+/4	-<br>3-/4	+<br>1+/1
c-mAb B9-1	++<br>3+/3	-<br>4-/4	-<br>2-/2	-<br>4-/4	-<br>4-/4	-<br>1-/1
c-mAb B3-9	+<br>3+/3	-<br>2-/3	-<br>3-/3	+<br>2+/3	-<br>3-/3	-<br>3-/3
$mAb_{(3-14)}$	+++<br>3+/3	-<br>3-/4	+<br>2+/2	+++<br>4+/4	+++<br>4+/4	-<br>1-/1
pAB	+++<br>3+/3	+++<br>4+/4	+++<br>2+/2	+++<br>4+/4	+++<br>4+/4	-<br>1-/1

.

Reference:

1. Köhler, G.; Milstein, C. Continuous cultures of fused cells secreting antibody of predefined specificity. *Nature* **1975**, *256*, 495–497, doi:10.1038/256495a0.
